# Longitudinal Change in Peripheral Quantitative Computed Tomography Assessment in Older Adults: The Hertfordshire Cohort Study

**DOI:** 10.1007/s00223-018-0442-0

**Published:** 2018-06-21

**Authors:** A. Patel, M. H. Edwards, K. A. Jameson, K. A. Ward, Nicholas Fuggle, C. Cooper, E. M. Dennison

**Affiliations:** 1 University Hospital Southampton NHS Foundation Trust, Southampton, UK; 2 MRC Lifecourse Epidemiology Unit, University of Southampton, Southampton, UK; 3 Portsmouth Hospitals NHS Trust, Portsmouth, UK; 4 MRC Elsie Widdowson Laboratory, Cambridge, UK; 5 NIHR Musculoskeletal Biomedical Research Unit, Nuffield Department of Orthopaedics, Rheumatology and Musculoskeletal Sciences, University of Oxford, Oxford, UK; 6 Victoria University, Wellington, New Zealand

**Keywords:** Peripheral quantitative computed tomography (pQCT), Epidemiology, Muscle, Bone mineral density, Bone parameters

## Abstract

There are few longitudinal data on change in bone structure and muscle mass, strength and function in later life. We report these, and consider bone–muscle interrelationships in older men and women. We studied 188 men and 166 women from the Hertfordshire Cohort Study, who underwent peripheral quantitative computed tomography (pQCT) of the radius and tibia in 2004–2005 and then again in 2011–2012. Grip strength and gait speed were also assessed at both timepoints. Percentage change per year was calculated for grip strength, gait speed, muscle cross-sectional area (mCSA), fat cross-sectional area (fCSA) and diaphyseal bone parameters [total area (Tt.Ar), cortical area (Ct.Ar), cortical density (cBMD) and trabecular density (tBMD)]. The mean (SD) age of men and women at baseline was 68.9 (2.5) and 69.2 (2.6) years, respectively. Rates of muscle area and strength loss did not differ by sex. Tt.Ar increased with age and faster in men [mean (SD) 1.78 (1.64) %/year] than women [mean (SD) 1.03 (1.69) %/year] in the radius (*p* < 0.001). In both the radius (*p* = 0.006) and tibia (*p* < 0.001), Ct.Ar reduced more rapidly in women than men. Change in Ct.Ar was associated with change in muscle area in the corresponding limb (radius; men: regression coefficient 0.36, 95% CI 0.20–0.52, *p* < 0.001; tibia; men: regression coefficient 0.14, 95% CI 0.00–0.27, *p* = 0.043, women: regression coefficient 0.16, 95% CI 0.01–0.30, *p* = 0.032). We have demonstrated that muscle strength and function decrease faster than muscle mass and have provided further evidence that changes in bone structure with age differ by sex.

**Electronic supplementary material:**

The online version of this article (10.1007/s00223-018-0442-0) contains supplementary material, which is available to authorized users.

## Introduction

In recent years, new techniques to assess bone mass and strength have been developed. One such technique is peripheral quantitative computer tomography (pQCT) [1]. This technique was introduced some years ago as a method for assessing 3D bone structure. In particular, it has the ability to measure volumetric bone mineral density (vBMD) and can estimate trabecular and cortical compartments of bone, as well as provide some information about the surrounding muscles [2]. With the above in mind, pQCT is thought to have the potential to capture novel aspects of bone and muscle geometry that may contribute to fracture risk; however, few longitudinal data are available for change in bone and muscle compartments using this technique.

The propensity to fracture is dependent upon the mechanical strength of a bone balanced against the forces it endures. Bones adapt to mechanical loads generated by mechanical usage, such as muscle contraction [3], implying the direct role of muscle on bone structure and strength. There are few longitudinal studies that have focused on associations between muscle measurements and changes in bone mineral content, density, organisation and structure [4–6]. Since mechanical loading preferentially increases bone size over mineral content and density [7], neglecting the assessment of bone structure may lead to an incomplete understanding of the influence of muscle loading on bone ageing. Furthermore, to date, few studies have investigated the relationship between muscle strength or physical performance and bone structure, and those investigating these associations often fail to consider potential confounding factors such as bone mass index, body size, diet and lifestyle [8–11]. We previously performed a cross-sectional analysis of the relationships between bone mass and strength with muscle mass, strength and function among participants from the Hertfordshire Cohort Study [12], reporting that muscle size was positively associated with radial bone size and strength in both sexes, with relationships persisting after adjustment for confounders. While grip strength showed similar associations with bone size and strength in both sexes, these were substantially attenuated after similar adjustment. Consistent relationships between gait speed and bone structure were not seen. In this study population, men had bigger muscles in both their upper and lower limbs, stronger grip strength and walked faster than women and as expected in both the radius and tibia, measures of bone size and density were higher in men than women.

Musculoskeletal ageing comprises loss of muscle mass and function, and perturbation of bone microarchitecture, resulting in increased propensity to fracture with low trauma, with its attendant associated morbidity and mortality. Therefore, the purpose of this longitudinal study was to build on previous work and document change in muscle size, strength and function and bone structure and mass with age, and to investigate the interrelationships between muscle size, strength or physical performance with changes in bone structure in a well-phenotyped cohort of older men and women.

## Methods

The Hertfordshire Cohort Study (HCS) is a population-based cohort study in the UK which was designed to examine the relationship between growth in infancy and the subsequent risk of adult disease. Study design and recruitment have been described in detail previously [13]. In brief, we traced men and women born between 1931 and 1939 in Hertfordshire and who still lived there in 1998–2003 when a nurse-administered questionnaire was carried out. In 2004–2005, 437 men and 447 women from the geographical area of East Hertfordshire were invited to a follow-up study to examine their musculoskeletal health. Of these, 322 men (65%) and 320 women (68%) agreed to participate. In 2011–2012, participants were invited to a second follow-up study to investigate change in their musculoskeletal health. Of the original 642 follow-up participants, 443 (222 men and 221 women) re-attended.

In 2004–2005, a detailed questionnaire was administered by a trained research nurse to gather information on lifestyle, medical history, cigarette smoking and alcohol consumption. In women, years since menopause and use of oestrogen replacement therapy were also obtained. A standardised activity score, ranging 0–100, was calculated based on responses to questions about the frequency of gardening, housework, climbing stairs and carrying loads in a typical week. Higher scores indicate a greater level of activity. Dietary calcium and socioeconomic status data had been collected at the initial questionnaire in 1998–2003. Height was measured to the nearest 0.1 cm and weight to the nearest 0.1 kg on a SECA floor scale (Chasmors Ltd, London, UK). Body mass index (BMI) was calculated as weight divided by height^2^ (kg/m^2^). Grip strength was measured three times in each hand using a Jamar hand-held isokinetic dynamometer using a standardised protocol [14]. The maximum value was used in analyses. Gait speed was quantified from the time taken to complete a 3-m walk test [15]. These anthropometric determinants were measured in 2004–2005 and again in 2011–2012.

In 2004-5, radial and tibial (non-dominant side) pQCT scans (Stratec 2000XL instrument, version 6.00) were performed on 276 men and 292 women. If the non-dominant side had previously sustained a fracture, the other side was scanned instead. During the second follow-up study in 2011–2012, 184 men and 166 women underwent repeat pQCT scans.

The radial length was measured from the distal end of the ulna styloid to the tip of the olecranon in millimetres (mm). The tibial length was measured from the prominence of the medial malleolus to the tibial plate (mm). Radial and tibial scout views identified measurement reference lines at the cortical end plates. Two slices were taken from the radial scan (4 and 66%), as well as forearm cross-sectional area of muscle (mCSA) and fat (fCSA). Three slices were taken from the tibial scan (4, 14 and 38%), as wSA and fCSA.. Muscle size analysis by pQCT has been found to be valid and reliable [16].

Trabecular parameters were measured distally (4% radius and 4% tibia) and cortical parameters were measured in the mid-shaft (radius, 66%; tibia, 14%) The following measurements were taken from the radius and tibia: total bone area (Tt.Ar), total mass, trabecular bone mineral density (tBMD), cortical bone mineral density (cBMD) and cortical bone area (Ct.Ar). Short-term measurement precision error ranged from 0.88% (total tibial density, 4% slice) to 8.8% (total radial area, 66% slice), but was typically between 1–3%. These figures were obtained by 20 volunteers who were part of the study undergoing two scans on the same day, with limb repositioning between examinations.

For all scans, a threshold of 280 mg/cm^3^ was used to separate the bone from the soft tissue background. Once separated, the default peeling algorithm was applied to the distal 4% scans to separate trabecular bone. With this peeling, 55% of the outer bone area was concentrically separated and defined as cortical and subcortical; the remaining 45% was defined as trabecular bone. For proximal scan locations, the default threshold of 710 mg/cm^3^ was used to separate cortical bone. Muscle CSA at the forearm and calf was derived using the default analysis steps that utilise various threshold and edge tracking settings to segment muscle from subcutaneous fat.

Ethical permission for the study was granted by the East and North Hertfordshire Ethical Committees. All participants gave written informed consent. Study participants who were taking drugs that are known to alter bone metabolism (e.g. Bisphosphonates) were excluded from the study. However, women who were taking hormone replacement therapy (HRT) were allowed to participate.

Variables were assessed for normality and transformed where necessary using the Fisher–Yates rank-based inverse normal transformation to create *z*-scores. Descriptive statistics for continuous variables were expressed as mean and standard deviation (SD) or median and interquartile range (IQR). Categorical variables were expressed as frequency and percentage (%). Differences between men and women were assessed using Student’s *t* tests, Mann–Whitney tests or Pearson’s *χ*^2^ tests, as appropriate. Linear regression analyses were used to examine the associations between longitudinal changes in muscle area and strength and change in bone structure over time. The regression analyses were undertaken with and without adjustment for the following lifestyle confounders: age, BMI, social class, smoker status, alcohol consumption, physical activity, dietary calcium intake and years since menopause and HRT use in women. All confounders were associated with at least one bone and muscle variable. None of the subjects were on bisphosphonate therapy at baseline but 41 subjects (9 men, 32 women) were receiving these therapies at follow-up; analyses were performed including and excluding these 41 people but excluding these people made little difference to the results. Similarly, we performed analyses including and excluding individuals on calcium or vitamin D supplements but again these adjustments made little difference to the results. Analyses were performed in men and women separately as we were interested in differing relationships between the two sexes, as has been observed in cross-sectional work [12]. However, we also tested for gender interactions; there was only one significant interaction (for change in CMA against tibial trabecular density). A *p* value of ≤ 0.05 was considered to be significant for all analysis. Statistical analyses were performed using STATA, version 14.

## Results

The baseline characteristics of the study population are displayed in Table 1; the mean age was 68.9 and 69.2 years in men and women, respectively. All women were postmenopausal. The mean body mass index score for women was 27.4 kg/m^2^, compared to 27.0 kg/m^2^ for men. Among men, 60.4% were either current or ex-smokers, compared with only 34.8% of women (*p* < 0.001). Men consumed more alcohol compared to women (*p* < 0.001). At baseline, men had larger muscles in both their upper and lower limbs (*p* < 0.001), stronger grip strength (*p* < 0.001) and walked faster than women (*p* = 0.010). In contrast, women had greater amounts of fat in their upper and lower limbs than men (*p* < 0.001). Additionally, at baseline, men had greater bone mass, density, size and strength in the radius, than women (*p* < 0.001). Similar sex-differences were found in the tibia bone parameters (*p* < 0.001).

**Table 1:** Baseline characteristics of the population

	Mean (SD)	Mean (SD)	
	Men (*n* = 184)	Women (*n* = 166)	*p* value
Age (years)	68.9 (2.5)	69.2 (2.6)	0.412
BMI (kg/m^2^)	27.0 (3.4)	27.4 (4.5)	0.333

Alcohol intake (units/week)	Median (IQR)	Median (IQR)	
	9.2 (2.8–17.8)	1.5 (0.0,4.8)	< 0.001

Smoker status	*N* (%)	*N* (%)	
Never	73 (39.7)	107 (65.2)	< 0.001
Ex	98 (53.3)	49 (29.9)	
Current	13 (7.1)	8 (4.9)	

Muscle parameters	Mean (SD)	Mean (SD)	
Forearm area (mm^2^)	4019 (506)	2564 (351)	< 0.001
Calf area (mm^2^)	8125 (1199)	6299 (954)	< 0.001
Grip strength (kg)	43.1 (8.0)	25.2 (6.0)	< 0.001
Gait speed (m/s)	0.94 (0.16)	0.89 (0.16)	0.010

Fat parameters	Mean (SD)	Mean (SD)	
Forearm fat area (mm^2^)	1020 (370)	1749 (720)	< 0.001
Calf fat area (mm^2^)	1718 (664)	3280 (1953)	< 0.001

	Mean (SD)	Mean (SD)	
Bone parameters (radius)			
4% total mass (mg mm^− 1^)	1.63 (0.25)	1.01 (0.18)	< 0.001
4% trabecular BMD (mg/cm^3^)	210 (37)	176 (44)	< 0.001
66% total area (mm^2^)	181 (26)	131 (20)	< 0.001
66% cortical area (mm^2^)	101 (14)	64 (11)	< 0.001
66% cortical BMD (mg/cm^3^)	1120 (36)	1099 (47)	< 0.001
Bone parameters (tibia)			
4% total mass (mg mm^− 1^)	4.24 (0.55)	2.97 (0.46)	< 0.001
4% trabecular BMD (mg/cm^3^)	241 (37)	222 (43)	< 0.001
14% total area (mm^2^)	575 (81)	472 (60)	< 0.001
14% cortical area (mm^2^)	201 (25)	141 (22)	< 0.001
14% cortical BMD (mg/cm^3^)	1101 (29)	1047 (50)	< 0.001

**Fig. 1:**
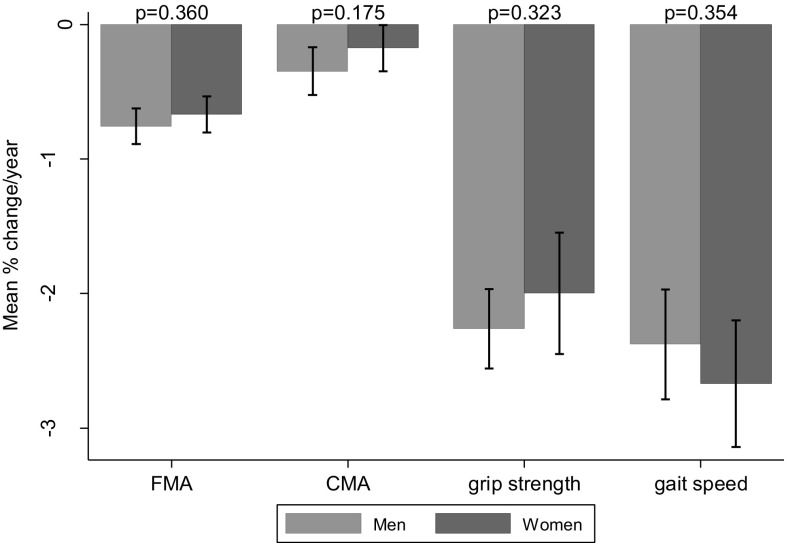
Percentage change in men and women of muscle area at the forearm (FMA) and calf (CMA); grip strength; gait speed

Longitudinal changes in muscle and bone parameters are summarised in Table 2, with change in muscle mass, strength and function also displayed in Fig. 1. Muscle strength and function decreased faster than muscle mass, as displayed in the figure. As expected, over time, men and women’s forearm and calf mCSA reduced. However, the rate of loss did not differ significantly by sex. In both sexes, the rate of loss of grip strength and gait speed was over 2% per year, whereas muscle size reduced at a slower rate. Figure 1 provides a graphical representation of the proportionate change in men and women of muscle area at the forearm and calf, grip strength and gait speed. The rate of increase in forearm and calf fCSA differed by sex, affecting men more than women [forearm; men: mean (SD) 1.25 (4.84) %/year, women: mean (SD) 0.17 (2.15) %/year, calf: men: mean (SD) 3.62 (20.5) %/year, women: mean (SD) 0.57 (2.65) %/year]. However, only the rate of increase in forearm fCSA reached statistical significance (*p* = 0.010).

**Table 2:** Change in muscle, fat and bone parameters with time in the cohort

Muscle parameters	Forearm			Calf		
	Men Mean (SD)	Women Mean (SD)	*p* value	Men Mean (SD)	Women Mean (SD)	*p* value
Muscle area (%/year)	− 0.75 (0.87)	− 0.67 (0.86)	0.360	− 0.34 (1.15)	− 0.17 (1.09)	0.175
Fat area (%/year)	1.25 (4.84)	0.17 (2.15)	0.010	3.62 (20.48)	0.57 (2.65)	0.069
Grip strength (%/year)	− 2.26 (2.02)	− 2.00 (2.94)	0.323			
Gait speed (%/year)				− 2.38 (2.73)	− 2.67 (2.96)	0.354
4% total mass (%/year)	− 0.50 (0.84)	− 0.69 (0.96)	0.055	− 0.40 (0.78)	− 0.59 (0.64)	0.015
4% trabecular BMD (%/year)	− 0.22 (1.08)	− 0.43 (1.25)	0.097	− 0.32 (0.77)	− 0.47 (0.75)	0.065
66/14% total area (%/year)	1.78 (1.64)	1.03 (1.69)	< 0.001	0.38 (0.41)	0.37 (0.30)	0.762
66/14% cortical area (%/year)	− 0.24 (0.92)	− 0.56 (1.16)	0.006	− 0.37 (0.70)	− 0.87 (1.08)	< 0.001
66/14% cortical BMD (%/year)	− 0.29 (0.28)	− 0.15 (0.39)	0.001	− 0.36 (0.24)	− 0.42 (0.37)	0.056

In both the radius and tibia, total bone mass and trabecular density tended to reduce at a greater rate in women than men (*p* < 0.10). Proximally, total area had a tendency to increase over time, showing positive values in both sexes. The rate of increase was significantly higher in men [mean (SD) 1.78 (1.64) %/year] than women [mean (SD) 1.03 (1.69) %/year] in the radius (*p* < 0.001). The rate of loss of cortical area was significantly greater in women than men in both the radius (*p* = 0.006) and tibia (*p* < 0.001). In the radius, cortical density decreased at a greater rate for men than for women (*p* = 0.001).

In general, changes in cortical bone geometry, not cortical BMD, were more strongly associated with changes in muscle mass and function, particularly at the radius (Table 3). In men, positive associations were seen between change in forearm muscle area and changes in radial total mass *z*-score (regression coefficient 0.30, 95% CI 0.14–0.47, *p* < 0.001) distally, radial Ct.Ar *z*-score (regression coefficient 0.36, 95% CI 0.20–0.52, *p* < 0.001) and cBMD *z*-score (regression coefficient 0.18, 95% CI 0.01–0.35, *p* = 0.034) in the mid-shaft, although the latter was not maintained after adjustment for demographic and lifestyle factors. No associations were seen in women. Change in calf muscle area was associated with changes in tibial Ct.Ar *z*-score, in both men (regression coefficient 0.14, 95% CI 0.00–0.27, *p* = 0.043) and women (regression coefficient 0.16, 95% CI 0.01–0.30, *p* = 0.032), though the association did not remain significant in men after adjustment for demographic and lifestyle factors. In women, there were also positive associations between change in calf muscle area and total mass *z*-score (regression coefficient 0.19, 95% CI 0.05–0.33, *p* = 0.007) and tBMD *z*-score (regression coefficient 0.25, 95% CI 0.12–0.39, *p* < 0.001).

**Table 3:** Interrelationships between change in muscle and bone parameters

	Change in forearm muscle area		Change in grip strength		Change in calf muscle area	
	Men	Women	Men	Women	Men	Women
Radius						
Change in total mass 4% site	0.30**	0.09	0.09*	0.02		
Change in trabecular density 4% site	0.12	0.03	0.03	0.01		
Change in total area 66% site	− 0.02	0.05	0.05	− 0.01		
Change in cortical area 66% site	0.36**	0.04	0.04	− 0.03		
Change in cortical density 66% site	0.18*	0.06	0.06	0.02		
Tibia						
Change in total mass 4% site			0.03	− 0.01	0.06	0.19*
Change in trabecular density 4% site			0.06*	− 0.02	− 0.02	0.25**
Change in total area 14% site			0.03	0.00	− 0.01	− 0.13
Change in cortical area 14% site			0.01	− 0.01	0.14*	0.16*
Change in cortical density 14% site			− 0.01	0.02	0.06	0.13

*p < 0.05, **p < 0.001

Positive associations were found between change in grip strength and change in forearm mCSA (*z*-score) in both sexes (men: regression coefficient 0.11, 95% CI 0.03–0.18, *p* = 0.005, women: regression coefficient: 0.06, 95% CI 0.01–0.11, *p* = 0.015). In women, change in gait speed was also associated with change in calf mCSA *z*-score (regression coefficient 0.07, 95% CI 0.01–0.12, *p* = 0.012). In both men and women, a change in gait speed was associated with a change in calf fCSA *z*-score (men: regression coefficient − 0.06, 95% CI − 0.12 to − 0.01, *p* = 0.029; women: regression coefficient − 0.05, 95% CI − 0.11 to − 0.00, *p* = 0.045), though this association did not remain in women after adjustment for demographic and lifestyle factors.

Change in grip strength was associated with change in total radial mass *z*-score in men (regression coefficient 0.09, 95% CI 0.01–0.16, *p* = 0.025) but not when demographic and lifestyle factors were adjusted for. Change in grip strength was not associated with any other changes in radial size or bone mineral density in either sex. In men, change in grip strength was associated with change in tibia diaphysis total area *z*-score (regression coefficient 0.10, 95% CI 0.02–0.17, *p* = 0.016) and tibial mass *z*-score but only when demographic and lifestyle factors were adjusted for (regression coefficient 0.10, 95% CI 0.02–0.19, *p* = 0.022). In contrast, no relationship was identified between change in grip strength and change in tibial size or density in women.

## Discussion

In this study, we report change in bone microarchitecture and muscle mass, strength and function with age in a community dwelling cohort of older adults. We have demonstrated that muscle strength and function decrease at a faster rate than muscle mass and furthermore have provided further evidence that changes in bone structure with age differ by sex. Cortical area consistently decreased more rapidly in women than men, whereas total bone area consistently increased more rapidly in men than women. In contrast, rates of loss of muscle parameters were similar in both sexes. Additionally, we have shown that there were positive associations between changes in muscle area and cortical area in both men and women. Finally, we have shown that changes in grip strength and gait speed were strongly associated with changes in muscle area in both sexes, and changes in bone size in men, but not women are in keeping with previous analyses [12].

The associations between changes both in muscle and bone size can be explained in several ways. First, the mechanostat hypothesis [3] states that bone adapts to changes in muscle in order to maintain appropriate strength to not fail during muscular loading. This is through activation of osteocytes which are able to sense strain; they either promote osteogenesis leading to bone formation or decrease bone resorption leading to reduced bone loss [17]. Second, we must acknowledge the various effects that genetic, non-mechanical (e.g. nutrition) and hormonal factors have on the musculoskeletal system, which can either directly or indirectly impact both muscle and bone development [18–21]. Third, physical activity can affect both muscle size and strength and bone structure. Finally, there is evidence supporting associations between birth weight and both bone health [22] and muscle function [23] in later life.

This study demonstrated that changes in grip strength and gait speed were strongly associated with changes in muscle size in both sexes, and changes in bone size in men, but not women. To our knowledge, the relationship between changes in physical performance as measured by grip strength and gait speed and changes in bone structure have not been previously investigated. However, a similar study, STRAMBO [24], defined physical performance by chair rises, static and dynamic balance and found that participants who scored poorly on one of the tests had significantly lower cortical bone area as assessed by high-resolution pQCT. These results are in keeping with our study’s finding although our assessment of physical performance employed different techniques.

Our analysis did not reveal consistent relationships between both changes in muscle area and strength and changes in bone mineral density (tBMD, cBMD) in weight-bearing and non-weight-bearing limbs. This is consistent with animal studies [25] that have shown cortical bone adapts its strength to mechanical loading by preferentially increasing bone size rather than bone mineral density, which is in keeping with our findings.

The main strength of this study was the population investigated. The individuals recruited were a non-selective community dwelling population who were born in Hertfordshire, and continued to live there at the age of 60–75 years. HCS characteristic and mortality patterns have previously been demonstrated to be similar to England [26]; thus generalisation to the wider population of older adults can be made. However, there are some limitations of this study. While not a true limitation, grip strength was taken as the highest of six measurements (three on the dominant side and three on the non-dominant side). Naturally, one would expect the maximum grip strength measurement to be recorded from the dominant side. Bone parameters were assessed on the non-dominant side, therefore this may attenuate the associations seen between grip strength and bone parameters to a degree. However, previous literature has reported the difference between grip strength in the dominant and non-dominant limbs to be small and relatively consistent [27]. Furthermore, the radius and tibia are not completely circular in cross section, therefore the manufacturer recommends the use of a circular ring model assumption in analysis of pQCT images. The alternative strategy would be to use an iterative contour detection method to provide a direct measurement, based on the true shape of the bone. However, because of the limited spatial resolution of pQCT, the pitfall in using an iterative approach is the failure to produce a measurement that can be utilised in an appreciable portion of individuals. It would be interesting to include body composition from DXA in our analyses but unfortunately these data were not available at the time points in question. Finally, we also recognise the limitations associated with self-reported physical activity, dietary calcium, smoking status and alcohol consumption.

In conclusion, we have demonstrated that changes in bone structure with age differ by sex. Additionally, we have shown that longitudinal changes in muscle size, in both upper and lower limbs, was strongly associated with longitudinal changes in bone size, in both men and women. This suggests that interventions to maintain muscle mass may help to ameliorate the age-related deterioration in bone health.

## Electronic Supplementary Material

Below is the link to the electronic supplementary material.

Supplementary material 1 (XLSX 66 KB)
